# Assessment of Potential Yield and Sustainable Management of Burbot, *Lota lota* (L.1758), in the Upper Heilongjiang River, China, Based on Population Parameters

**DOI:** 10.3390/biology14030248

**Published:** 2025-02-28

**Authors:** Wanqiao Lu, Zepeng Zhang, Hongyu Jin, Huili Shao, Shenhui Li, Yue Xing, Lei Li

**Affiliations:** 1 Heilongjiang River Fisheries Research Institute, Chinese Academy of Fisheries Sciences, Harbin 150070, China; luwanqiao@hrfri.ac.cn (W.L.); zhangzepeng@hrfri.ac.cn (Z.Z.); jinhongyu@hrfri.ac.cn (H.J.); 14415156808538@163.com (H.S.); muqi1300@outlook.com (S.L.); xy13027563833@163.com (Y.X.); 2 Scientific Observing and Experimental Station of Fishery Resources and Environment in Heilongjiang River Basin, Ministry of Agriculture and Rural Affairs, Harbin 150070, China

**Keywords:** *Lota lota*, ecosystem, FISAT II, population status, resource utilization, Heilongjiang River

## Abstract

We evaluated growth, mortality, and resource utilization of burbot, *Lota lota*, in the upper reaches of Heilongjiang River, China. The population has experienced significant decline, and, despite substantial recovery, concerns regarding resource management have arisen. A total of 638 specimens collected from October 2022 to October 2023 were used to calculate the relationship between body length and weight, which ranged from 107 to 529 mm and from 8.91 to 1474.17 g, respectively. FAO FiSAT II software was used to estimate population parameters based on body length frequency distribution of the samples. The calculated progressive body length value and the growth coefficient were 551.25 mm and 0.16, respectively. The natural mortality rate of burbot is 0.31 a^−1^. Assuming a total mortality rate of 0.41 a^−1^, the estimated current exploitation rate is 24.39%. Total biomass (94.76 t) and maximum sustainable yield (14.69 t) were estimated based on length-structured virtual population analysis. Burbot, of the upper reaches of Heilongjiang River, is a fast-growing fish that is well-suited to the ecology of the investigated area. The current research yielded suggestions for utilization of resources to ensure the preservation of the species, as well as the development of the local economy.

## Simple Summary

As the only freshwater species of Gadidae in the world, burbot, *Lota lota*, has a unique biological value. The uncontrolled utilization of burbot without knowing the current status of the stock will have a great impact on the whole inland cold-water fish community structure and ecosystem. We estimated and analyzed the data from our fieldwork in combination with mathematical modeling. We found that the burbot population in the upper Heilongjiang River has been seriously damaged. However, with the protection policies introduced by the local governmental departments in the past 10 years, the population has recovered considerably. Although the population has not recovered to its optimal state, we believe that it is appropriate to control the fishing size and the annual catch yield to more than 251 mm and less than 14.69 tons, respectively.

## 1. Introduction

Burbot, *Lota lota*, is the only freshwater species of Gadidae and an economically important cold-water fish [1,2]. In addition, burbot is also one of the only two circumpolar fish species in the world, distributed in Asia, Europe, and North America. It has high nutritional [3] and economic value [4] and is one of the most frequently consumed species worldwide [1]. It is highly tolerant to cold [5] and capable of spawning beneath ice [6], and it is widely found in rivers and lakes at latitudes above 45° N, such as Lake Michigan [7], Lake Hovsgol [8], and Heilongjiang River [9]. The Heilongjiang River, home to over 100 species of fish [10], represents its easternmost distribution. The upper reaches of the Heilongjiang River have a long period of ice cover, requiring cold-water tolerance of resident fish.

As a typical carnivore, burbot has compact meat with high protein and low-fat content [11]. Its large liver has high nutritional value and is used in making fish liver-oil dietary supplements [4]. As a result, burbot once held an important position in the fishing industry in North America [5,6]. Burbot muscle contains seven amino acids and the unsaturated fatty acids DHA and EPA, essential for humans [1,3]. Burbot has a high content of type III antifreeze protein useful in the manufacture of cold- and frost-resistant materials [1,4]. Overall, burbot not only tastes extremely delicious but also is rich in nutrition. Therefore, burbots garner high attention and popularity in the local market. And this also leads to a significantly higher market value for burbots compared to other freshwater fish. However, the reproductive yield of wild burbot is low [12,13,14], and the population has declined in multiple areas [2]. The study of burbot has primarily focused on aspects of growth [15,16], reproduction [17], and diet [2,16], as well as aquaculture technology [1,18,19], molecular biology [20], and disease [21,22]. In history, burbot was once one of the most important economic fish in the region. However, due to overfishing, the burbot population has been sharply reduced. As a result, burbot was listed as a rare and precious aquatic wildlife in China in 2015. During the following decade, the local wildlife protection department and the government introduced a series of conservation measures, including but not limited to setting up nature reserves, targeted fishing bans, and publicity. These measures ensure the living space of various fish species, including burbots. The burbot population resources have recovered in recent years.

In fact, effective evaluation research holds significant importance for protecting fish resource populations and optimizing fish community structure, thereby contributing to the stability of ecosystems. Likewise, the evaluation results offer valuable scientific insights for the sustainable utilization of fish population resources. However, there are few reports on the population parameters and resource status of burbots. Therefore, at the request of local wildlife authorities, we conducted a detailed analysis, including size frequency distribution, length–weight relationship, and mortality rate, to determine the population characteristics and resource status of burbot in the upper reaches of Heilongjiang River, China. Our research findings will provide important guidance for the local fishing industry and allocation of quotas.

## 2. Materials and Methods

### 2.1. Study Area and Sample Collection

**Figure 1:**
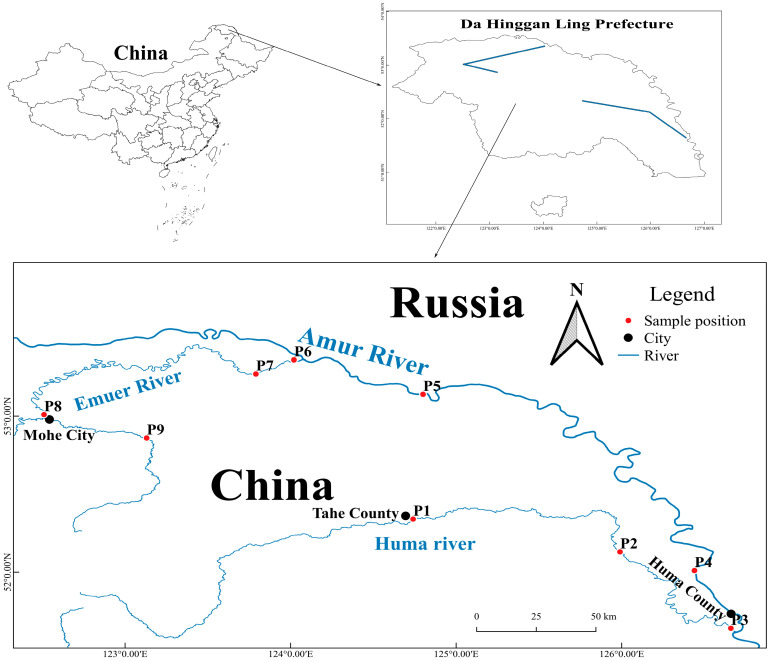
Study area.

According to historical data, the length of burbot in the Heilongjiang basin has been as much as 1500 mm [13]. However, it is the common phenomenon that burbot grows to about 300 mm. Population recruitment is available when burbot grows to 3–4 years of age. Burbot is a typical cold-water fish. The spawning time of burbot population is mainly concentrated from November of this year to March of the next year. So, according to the living habits and historical survey data of burbot, combined with the needs of the local fishery administration department for fish resource management, four positions were set up in the Emuer River, three positions in the Huma River, and two positions in the Heilongjiang Main Stream (Figure 1). The sampling period is from October 2022 to October 2023. The sampling frequency is at least once per season. In general, we can reasonably assume that the size structure and population density are the same throughout the sampled area. So, we employed commercial fishermen (same fishermen, same nets) to collect 638 burbot specimens from the study area (Figure 1). Gill nets were 40–60 m long and 1.2–1.5 m high, with mesh sizes of 1, 2, 4, 6, 8, and 10 cm. Ground cages were 25 m long, 50 cm wide, and 50 cm high, with 0.5 cm mesh. The size of the net was measured by straightening the opposite knot. Meanwhile, samples were also collected every 12 h. The nets were then reintroduced back into the water. The measured data included body length, fork length, total length, and body weight. Specifically, body length represents the straight-line length from the snout end to the base of the tail fin of a sample. Meanwhile, weight represents the total weight of the sample. The measuring accuracy of the ruler and scale used for measurement is 1 mm and 0.01 g, respectively. And that was all performed by professional researchers [23].

### 2.2. Data Analysis and Processing

#### 2.2.1. Size Characteristics and Parameters

The total number of burbot samples that can be used for data analysis in this study was 638. We measured and weighed fish immediately after capture. On the premise that the relationship between body length (*L*) and body weight (*W*) conforms to the power function curve [24], the length–weight relationship was calculated as

$$W=a\cdot L^{b}$$

where *W* is body weight (g), *L* is body length (mm), *a* is condition factor, and *b* is a power exponent coefficient. Both *a* and *b* are derived from fitting a power function of body *L* to *W*. Using the von Bertalanffy growth formula (VBGF) [25], *L* is calculated as

$$L_{t}=L_{\infty }[1-e^{-k(t-t_{0})}]$$

where $L_{t}$ is the hypothetical length at age *t*, $L_{\infty }$ is the asymptotic length of the sample, *k* is the average curvature of the sample growth curve, *t* is the age of the individual sample estimated from *L*, and $t_{0}$ is the hypothetical age at which length is zero [26]. Combined with the distribution of body length frequency, the ELEFAN program in FiSAT II software was used to estimate $L_{\infty }$, *k*, and $t_{0}\;$ used in the VBGF. And based on *L* and *W*, taking the derivative of *t*, we can obtain equations for the instantaneous growth rate using the following formulae [27,28,29]:

$$\frac{dLt}{dt}=k\times L_{\infty }\times e^{\left(-k\left(t-t_{0}\right)\right)}$$

$$\frac{dWt}{dt}=b\times k\times W_{\infty }\times e^{\left(-k\left(t-t_{0}\right)\right)}{\left[1-e^{\left(-k\left(t-t_{0}\right)\right)}\right]}^{b-1}$$

Estimates of $L_{\infty }$ and *k* were used to calculate the growth performance index ($\varphi^{\prime }\;$) and growth inflection point age ($t_{tp}$) [30] using the following formulae:

$$\varphi^{\prime }=lgk+2lgL_{\infty }$$

$$t_{tp}=\frac{lnb}{k}+t_{0}$$

#### 2.2.2. Mortality, Exploitation, and Survival

The total instantaneous mortality rate (*Z*) was estimated using a length-converted catch curve method in FiSAT II software [31], expressed as

$$ln\left(\frac{N}{△t}\right)=-Zt+c$$

where *N* is the number of burbots in different length groups; △t is the time required for the corresponding body length group to go from the lower limit to the upper limit; $ln\left(\frac{N}{△t}\right)$ is the number of deaths in the population, *N*, at time *t*; and *c* is the intercept.

The instantaneous rate of natural mortality (*M*) was obtained from the empirical relationship of Pauly’s equation, expressed as

$$\ln M=-0.0066-0.279lnL_{\infty }+0.6543\ln k+0.4634\ln T$$

where *T* is the annual mean temperature (°C) of the target habitat. According to data obtained from relevant local authorities, *T* is 12 °C.

Based on *Z* and *M*, *F* (instantaneous rate of fishing mortality) can be found using the following formula. The exploitation rate (*E*) [32] and survival rate (*S*) are represented by the relationship between the following three instantaneous mortality rates [33], where e is Euler’s number.

F=Z−M

$$E=\frac{F}{Z}\times 100\%$$

$$S=e^{-Z}$$

The critical age of a population refers to the age at which the individual instantaneous natural mortality rate equals the growth rate in *W*, or when the biomass of a generation of organisms reaches its maximum. Assuming that the weight growth of each individual in the same generation of burbot follows the von Bertalanffy growth equation, this can be expressed as

$$\frac{dWt}{\left(Wt\times dt\right)}\approx \frac{dB}{\left(B\times dt\right)}=M$$

where *B* is the biomass of burbot in the upper reaches of Heilongjiang River.

#### 2.2.3. Survival Probability

The survival probability for burbot in the upper reaches of Heilongjiang River was estimated by using the length-based Bayesian biomass method in FISAT II software. Some numerical values were taken from the Markov-chain Monte Carlo formula [34]:

$$N_{L}=N_{L_{start}}{\left(\frac{L_{\infty }-L}{L_{\infty }-L_{start}}\right)}^{\frac{Z}{k}}$$

where *L_start_* is the opening catch *L*, and *N_L_* is the number of surviving captured individuals of length, *L*. All individuals entering the net are retained in the net. The body length frequency data do not contain any information about absolute abundance. Therefore, when both sides of the equation are divided by their respective sums, the equation remains unchanged [34,35]. The formula can be expressed as follows:

$$\frac{N_{L}}{\sum N_{L}}=\frac{{\left(\frac{L_{\infty }-L}{L_{\infty }-L_{start}}\right)}^{\frac{Z}{k}}}{\sum {\left(\frac{L_{\infty }-L}{L_{\infty }-L_{start}}\right)}^{\frac{Z}{k}}}$$

Under the premise of no commercial fishing, the parameters in the formula do not provide information of abundance. Therefore, when F = 0, Z/k is M/k, $L_{start}$ is 0, and $N_{L_{start}}$ can be set to 1. We can use mathematics to calculate the rate of survival of individual burbot to length, *L* [35]:

$$P_{L\to L_{\infty }}={\left(1-\frac{L}{L_{\infty }}\right)}^{\frac{M}{k}}$$

#### 2.2.4. Catch per Unit Replenishment

The improved Beverton–Holt (B-H) dynamic model formula was used to analyze the relative yield per recruit (*Y′/R*) [36] as

$$Y^{\prime }/R={EU}^{M/k}\left[1-\frac{3U}{1+m}+\frac{3U^{2}}{1+2m}-\frac{U^{3}}{1+3m}\right]$$

$$(U=1-\frac{L_{c}}{L_{\infty }},\;m=\frac{k}{Z})$$

where *L*_c_ is the opening catch length, and *E* is the exploitation rate.

#### 2.2.5. Resources

Based on *L* distribution data of the burbot samples, resource quantity is calculated by length-structured virtual population analysis [33,37]:

$$N_{t}=C_{t}\cdot \frac{M+F_{t}}{F_{t}}$$

$$C_{i}=N_{i+\Delta t}\cdot (\frac{F_{i}}{M+F_{i}})\cdot (e^{\left(M+F_{i}\right)\Delta t_{i}}-1)\;(\Delta t_{i}=(t_{i+1}-t_{i},t_{i}=t_{0}-\frac{1}{k}\cdot \ln(1-\frac{L_{i}}{L_{\infty }}))$$

$$N_{i}=N_{i+\Delta t}\cdot e^{\left(M+F_{i}\right)}$$

where *N_t_* is the number of burbots of maximum length in the study area; *C_t_* is the number of burbots of maximum length; *F_t_* is the instantaneous mortality of burbots of maximum length in the target area; and *N_i_* and *N_i+_*_Δ*t*_ are the resources of burbots at age *I* and *i* + Δ*t* age, respectively. *C_i_* is the catch quantity of burbot at age *i*. *F_i_* is instantaneous fishing mortality of burbot at age *i* in the target area.

#### 2.2.6. Data Analysis

The FISAT-II and Microsoft Excel were employed for statistical analyses [31]. ArcGIS 10.5 software was used to create the sampling-station map.

## 3. Results

### 3.1. Biometric Characteristics of Burbot

**Figure 2:**
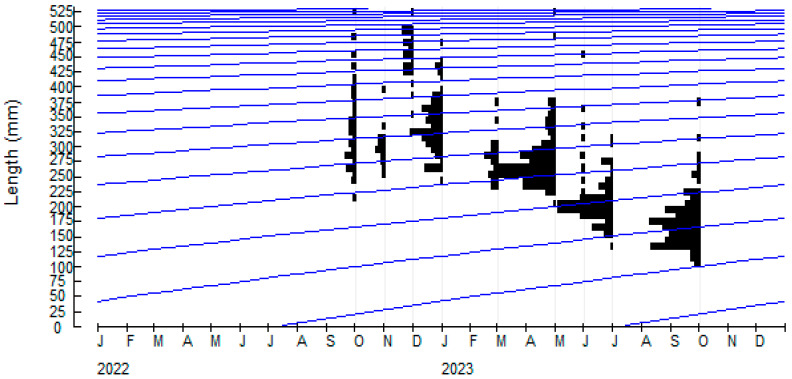
Growth curve of burbot in the study area by ELEFAN superimposed on the restructured length-frequency diagram ($L_{\infty }$ = 551.25 mm and *k* = 0.16). The black sections represent the number of different body length groups in the burbot population (frequency distribution of body length). The purple curve is the growth curve of burbot population (*k*).

**Figure 3:**
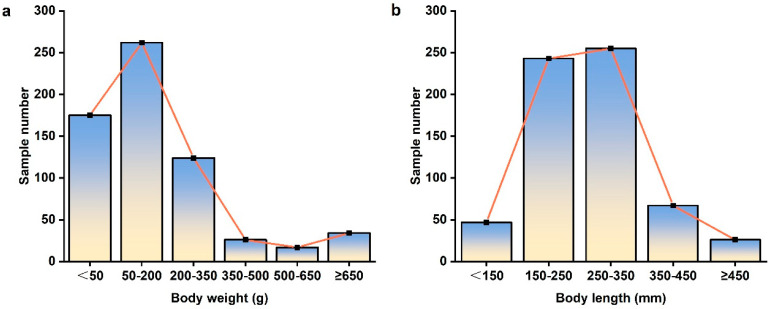
Body length − body weight distribution of burbot collected from the study area. (**a**) Body weight (g) distribution; (**b**) Body length (mm) distribution.

**Figure 4:**
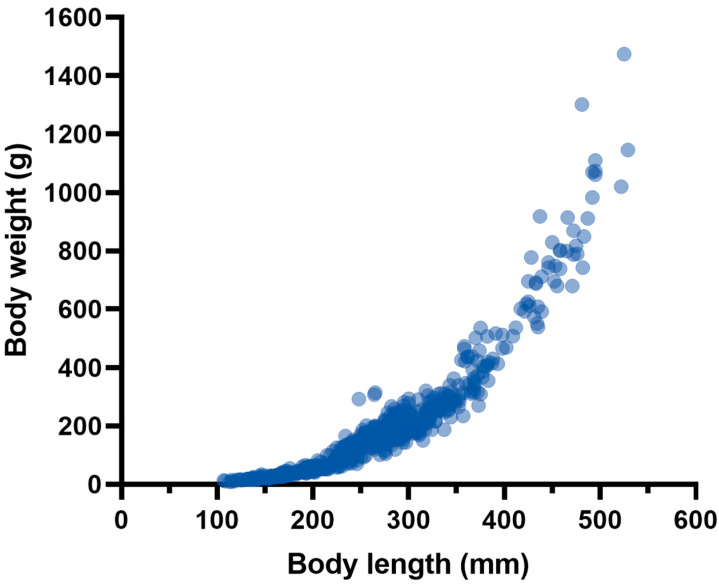
Length–weight relationship of burbot collected from the study area.

Six-hundred and thirty-eight burbots were collected from the upper reaches of Heilongjiang River (Figure 2). The quantity of samples collected at sampling positions is shown in Table 1. Body length ranged from 107 to 529 mm, and body weight from 8.91 to 1474.17 g (Figure 3). Mean *L* and *W* were 261.69 mm and 189.68 g, respectively (Table 2). Similarly, *a* and *b* also can be estimated, respectively. Therefore, the relationship between *L* and *W* weight of burbot was $W_{t}=4\times 10^{-6}Lt^{3.135}$(*R*^2^ = 0.9544) (Figure 4).

### 3.2. Population

#### 3.2.1. Growth Parameters

The progressive length and growth constant of burbot are 551.25 mm and 0.16, respectively. The growth performance index ($\varphi^{\prime }\;$) is estimated to be 4.69 (Table 2). Age at zero length ($t_{0}$) was found to be −0.7968 (Table 2). The $W_{\infty }$ was used to estimated to be 1571.04 g. Body length and weight was calculated as $L_{t}=551.25\times \left(1-e^{\left(-0.16\left(t+0.7968\right)\right)}\right)$ and $W_{t}=1571.04\times {\left(1-e^{\left(-0.16\left(t+0.7968\right)\right)}\right)}^{3.135}$, respectively.

#### 3.2.2. Inflection Point of Weight Growth

**Figure 5:**
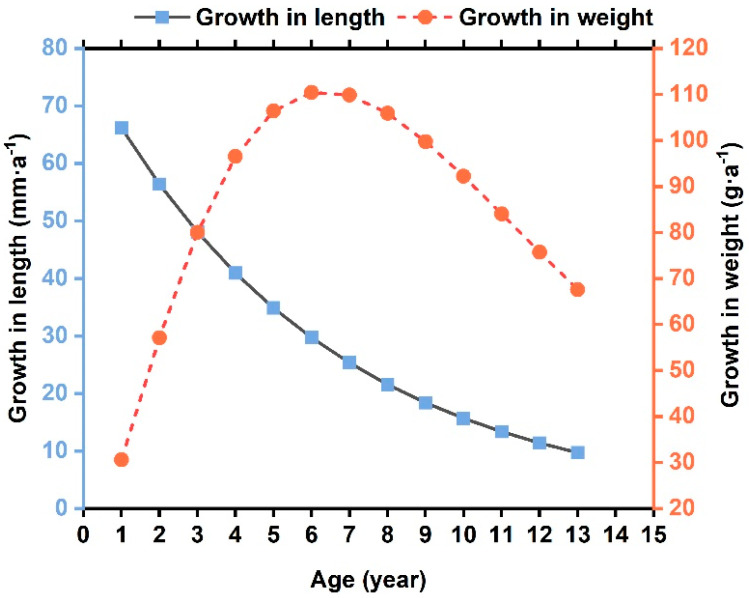
Growth curves of burbot body length and body weight estimated by von Bertalanffy growth model in the study area.

According to the above results, the equations for the instantaneous growth and weight rate are $88.20e^{\left(-0.16\left(t+0.7968\right)\right)}$ and $788.03\times e^{\left(-0.16\left(t+0.7968\right)\right)}{\left[1-e^{\left(-0.16\left(t+0.7968\right)\right)}\right]}^{2.135}$, respectively (Figure 5). The rate of *L* growth decreases with age. Body weight initially rises and then falls, with the peak referred to as the inflection point age ($t_{tp}$). When $\frac{d^{2}Wt}{dt^{2}}=0$, the $t_{tp}$ of burbot is 6.34 a (Table 2). Body length and weight at $t_{tp}\;$ are 375.28 mm and 470.63 g, respectively. This is basically consistent with the results identified by Ms. Shao using age data [2,13].

### 3.3. Mortality and Exploitation Rate

#### 3.3.1. Estimate of Mortality

**Figure 6:**
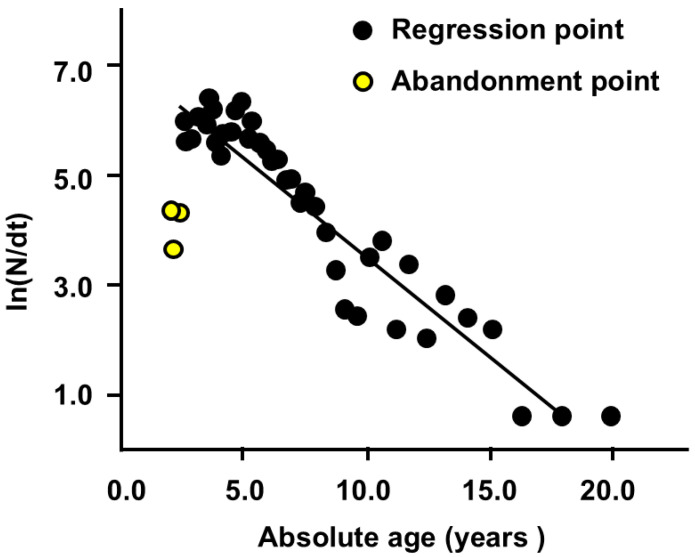
Length-converted catch curves of burbot in the study area. The slope of the line represents the total instantaneous mortality rate (*Z*) of the burbot population.

Typically, we can calculate three instantaneous mortality rates for burbot populations: The total instantaneous mortality (*Z*) can be estimated using the *L*-converted catch curve method in FiSAT II software (Figure 6). The slope of the line produced by the formula $In\left(\frac{N}{△t}\right)=-0.41t+6.25$ (r = −0.91) (Figure 6) represents the estimated value of *Z*, 0.41 a^−1^ (Table 2). On the premise that the water temperature (T) of the burbot habitat averaged 12 throughout the year, the value of *M*, *F*, and exploitation rate (*E*) was estimated to be 0.31 a^−1^, 0.10 a^−1^, and 24.39%, respectively (Table 2).

Using the length-based Bayesian biomass method, the probability of burbot survival to ages 1 through 7 years in the upper reaches of Heilongjiang River was estimated as 42.02%, 30.82%, 22.60%, 16.58%, 12.16%, 8.92%, and 6.54%.

#### 3.3.2. Critical Age of Burbot

The critical age is the age at which the instantaneous natural mortality rate of an individual is the same as the growth rate when there is no fishing pressure. In other words, the age at which the biomass of a generation of organisms reaches its maximum. The formula is $\frac{dWt}{\left(Wt\times dt\right)}\approx \frac{dB}{\left(B\times dt\right)}=M$. The estimated critical age of burbot is 5.05 a (Table 2). The corresponding *L* and *W* are 334.94 mm and 329.49 g, respectively.

### 3.4. Resource Status

**Figure 7:**
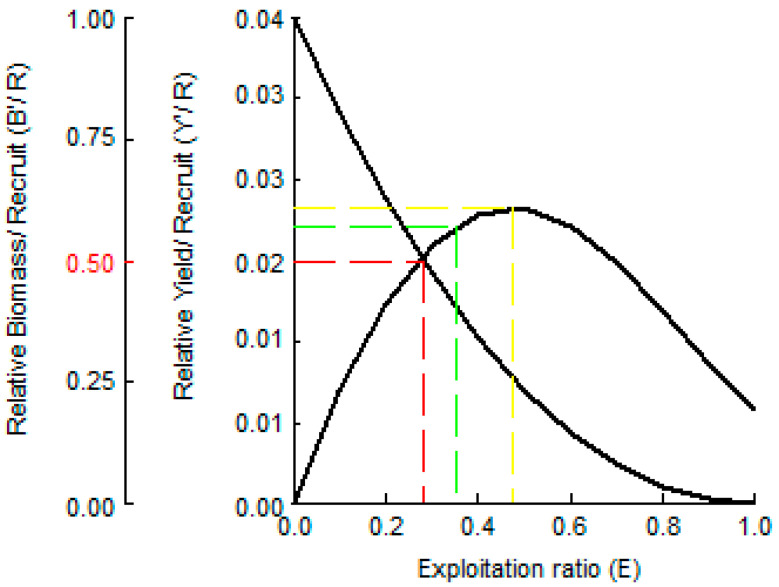
Two-dimensional analysis for *Y′/R* and *B′/R* of burbot in the study area. *Lc* = 130 mm. The red line represents *E*_50%_, The green line represents *E*_0.1_, The yollow line represents *E_ma_*.

**Figure 8:**
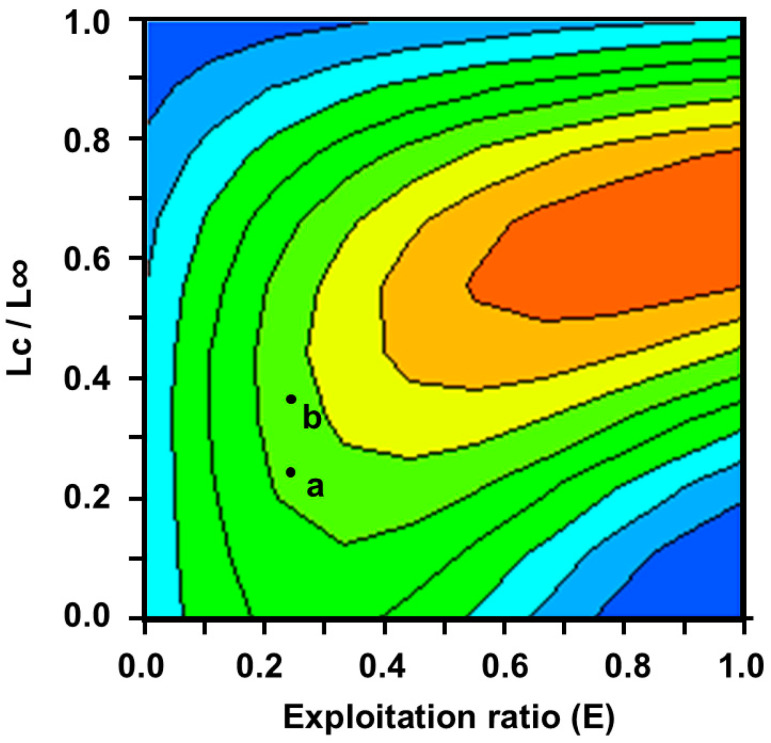
Relationship between relative yield per recruit, *Y′*/*R*; exploitation rate, *E*; and catchable size of burbot in the study area. The lines in the figure are contour lines of *Y′/R* values. a represents the *Y′/R* value of the current state. b represents the state with the highest *Y′/R* value under the premise of unchanged development rate in the current state.

The opening catch, *Lc*, of burbot was 130 mm (Table 2). Specimens ≤ 130 mm can be considered a supplementary population. This is one of the most important premises for analyzing the relationship between the relative yield per recruit (*Y’/R*) and *E* (Figure 7). Under current conditions, the value of $Y^{\prime }/R$ is 0.0185 (Figure 7, point a). With an increase in specification of the opening catch, biomass per recruit ($B^{\prime }/R$) steadily decreases, and relative yield per recruit, $Y^{\prime }/R$, initially increases and then subsequently decreases. The peak is the value of $Y^{\prime }/R$ at *Lc*, 198.45 mm (~0.0193) (Figure 7, point b). On the other hand, with an increase in fishing pressure, biomass per recruit ($B^{\prime }/R$) steadily decreases, and relative yield per recruit $Y^{\prime }/R$ initially increases and then subsequently decreases. The presence of an inflection point, *E_max_*, is apparent. Exploitation rates, including *E*_0.1_, *E*_50%_, and *E_max_*, of burbot were obtained by the knife-edge selection hypothesis in the B-H dynamic equation as 0.35, 0.28, and 0.47, respectively (Table 2). The dynamic change trend among the yield per unit replenishment, *L_c_*, and *E* was estimated (Figure 8). Under current fishing conditions, the values of $L_{C}/L_{\infty }$ and E are 0.24 and 0.16, respectively.

**Figure 9:**
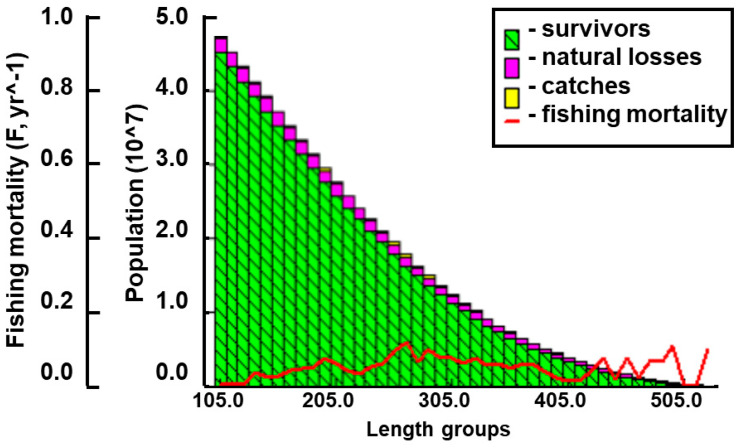
Length-structured virtual population analysis of burbot in the study area.

We obtained the annual fishing yield of the survey area from local authorities. Combining length-structured VPA and FISAT II software, we estimated the quantity and biomass of the burbot population in the upper reaches of Heilongjiang River as 273.40 thousand fish and 94.76 t (Table 2 and Table 3) (Figure 9).

## 4. Discussion

### 4.1. Current Status of Burbot Population

Body length and weight, growth rate inflection point, mortality rate, and exploitation rate were estimated and applied to further analysis. As important parameters of the population, the asymptotic body length, growth ease parameters *a* and *b*, and *k*-values not only respond to the growth dynamics of the target fish population but also laterally respond to its population life history [37,38]. Excluding the influence of gear selection, fishing ability, and environmental conditions, it is reasonable to assume that the number of burbot in the sampling area with *L* outside this range is negligible, especially among large individuals. In our data, the asymptote length, $L_{\infty }$, was 551.25 mm. According to historical data, the length of burbot in the Heilongjiang basin has been as much as 1500 mm [13]. Meanwhile, the results of this study are basically consistent with the results reported by Shaw [8] in Lake Hovsgol, which is roughly at the same latitude as Heilongjiang River. It is worth mentioning that the $L_{\infty }$ value of burbot in Lake Hovsgol is 880 mm, which is higher than the value in this study. On the contrary, the *k* value of 0.14 is smaller than the results of this study. Our data indicated that burbots from the upper part of the Heilongjiang River grew faster than those from Lake Hovsgol, even though the growth potential of burbots from the upper part of the Heilongjiang River was less than that of Lake Hovsgol. Consequently, without considering the impact of habitat and feeding on ecological factors, it is plausible to infer that the population has been affected by the fishery. In terms of growth characteristics, the growth performance coefficient, $\varphi^{\prime }$, was estimated to be 4.6. It provides the possibility for a recovery of the population.

A surprising result was the estimated annual exploitation rate of only 24.39% for burbot, much lower than the Gulland’s Law 50.00% [39] and the maximum exploitation rate, *E_max_* [40]. Based on these results, the *Z*/*k* value for the burbot population is 2.56, implying that the population is currently undergoing a high mortality rate [37]. We used the natural mortality (*M*) and growth parameter (*k*) for further validation of the data. The *M*/*k* value is 1.94, which is within 1.5–2.5, the normal range [41].

Burbot was formerly included in the list of China’s endangered fish species and grade II protected animals [5,41]. This implies that the burbot population in the upper Heilongjiang River in China was subjected to high fishing pressure, leading to the decline of its population. Subsequently, local departments and the government established natural reserves and implemented policies such as restricted fishing to ensure the living space of various fish species, including burbot. Firstly, the reduction of fishing pressure has a significant and intuitive effect on the recovery of burbot resources. At the same time, this also protects the local fish community structure and provides assistance in obtaining prey for burbot. Secondly, the establishment of nature reserves provides a foundation for the purification of water quality and the stability of the ecological environment. This provides good protection for the spawning grounds and habitats of burbot. All of these factors have led to the recovery of burbot resources, while also ensuring that burbots are no longer listed as dangerous. The population has not reached the established target [5,42], but it has recovered sufficiently to support a managed fishery.

### 4.2. Rational Utilization and Sustainable Management of Burbot

Fish resource utilization has to be based on fishery data and controlled exploitation. Fishery data can be roughly categorized as individual growth potential, population size [33], reproduction [43], and yield [41]. The indicators of individual growth potential and population control are the inflection age and critical age. With respect to reproduction, studies have shown that harvest should be controlled after the age of first sexual maturity [44], which, in burbot, is generally three years [1,2] and corresponds to *L* of 250.98 mm. Using the improved B-H dynamic model, the $Y^{\prime }/R$ values of the mentioned indicators were estimated under current fishing pressure. With consistent fishing pressure producing change in *L_c_*, $Y^{\prime }/R$ increases to an inflection point and subsequently decreases. The peak is the value of $Y^{\prime }/R$ at *L_c_*, 198.45 mm (~0.0193). Since the current burbot population is likely under-fished, and resource management consists mainly of conservation, we only estimated the $Y^{\prime }/R$ values and the inflection point under consistent fishing pressure. Therefore, only the $Y^{\prime }/R$ values for individual growth potential, population control, and reproductive stage were estimated: 0.0125, 0.0152, and 0.0187 respectively. The $Y^{\prime }/R$ value for reproductive stage is slightly greater than the peak $Y^{\prime }/R$ value, which can effectively protect the supplemental population of a given year. The individual growth potential and population control may be more suitable for burbot than the value of $Y^{\prime }/R$, since the body length cited in the maximum $Y^{\prime }/R$ value is too small to effectively protect the supplementary population of burbot. Limiting the catch size to 250.98 mm is optimal. However, in actual operations, it is difficult to control the fishing accuracy within 250.98 mm. Therefore, considering the actual demand and the healthy development of the burbot population, the fishing specification should be controlled above 251mm.

Controlling fishing yield involves choosing the optimal biological reference point, such as *F*_0.1_ or *F*_msy_ [43]. Considering the current state of the burbot population in the upper Heilongjiang River, the introduction of sustainable fishing operations is urgent; hence, *F*_msy_ was chosen as the biological reference point in this study. Assuming that the resource biomass is the biomass at the time that the maximum sustainable yield is obtained ($M=1/2B_{o}$) based on the equations $F_{msy}=1/2B_{o}$, $B_{msy}=M$, hence${\;MSY=B}_{msy}\times F_{msy}=0.5\times M\times B_{o}=0.5\times 0.31\times 94.76=14.69t$ [45], the maximum sustainable annual catch is 14.69 t. Meanwhile, it is also necessary for fishermen to write fishing diaries during fishing operations. This not only plays a substantial role in the restoration of burbot resources, but it also provides reference for the enforcement standards of relevant departments. In addition, as a typical cold-water fish, burbot is highly sensitive to changes in water temperature. However, in the context of global warming, the high-quality habitats available for the survival of burbot are gradually decreasing. Overall, the restoration of burbot resources in the future will be a long-term effort.

Rational utilization of resources is based on ecological sustainability. According to the local consulting survey, burbot is usually sold at about 80 CNY/kg. If fishing operations are carried out in accordance with the above strategy, the rational utilization of fishery resources is realized from an ecological standpoint. This level of yield can increase the annual income of local people by about CNY 1.18 million.

## 5. Conclusions

The population of burbot in the upper Heilongjiang River had been depleted, and, after a period of conservation, it has shown significant recovery. Although it has not yet recovered to its optimal state, it shows signs of being underexploited. We suggest that the catch should be limited to fish greater than 251 mm in length, and the annual harvest to 14.69 t, and in addition to the consistency of the population decline with the previous researchers. These regulations will ensure the preservation of the species, as well as the development of the local economy. In addition, it is also necessary to form a rigorous fishing diary with scale enforcement, which can provide reference and guarantee for future data accumulation. At the same time, we will consider a variety of estimation methods, including traditional mathematical models, such as e-DNA, etc., to ensure the accuracy and timeliness of the estimation results.

## Figures and Tables

**Table 1:** Sample distribution in positions.

Position	P1	P2	P3	P4	P5	P6	P7	P8	P9
Quantity	94	68	37	72	81	57	45	103	81

P represents the sampling position. Quantity is the number of samplings in each station.

**Table 2:** Growth and reproduction parameters ($L_{\infty }$, $\varphi^{\prime }$, *t*_0_, *a*, *b*, and *k*), mortality (*Z*, *M*, and *F*), and fishery parameters (*E*, and *Lc*) of burbot in the study area.

Parameter	Value
Mean body length ^1^	261.69 ± 85.55 mm
Mean body weight ^2^	189.68 ± 207.26 g
$\mathrm{The}\;\mathrm{asymptotic}\;\mathrm{length}\;(L_{\infty }$) ^1^	551.25 mm
The growth constant (*k*)	0.16
$\mathrm{Growth}\;\mathrm{performance}\;\mathrm{indices}\;(\varphi^{\prime })$	4.69
$\mathrm{Age}\;\mathrm{at}\;\mathrm{zero}\;\mathrm{length}\;(t_{0}$)	−0.7968
$\mathrm{Inflection}\;\mathrm{point}\;\mathrm{age}\;(t_{tp}$) ^3^	6.34 a
Total instantaneous mortality (*Z*) ^3^	0.41 a^−1^
Nature instantaneous mortality (*M*) ^3^	0.31 a^−1^
Fishing instantaneous mortality (*F*) ^3^	0.10 a^−1^
Exploitation rate (*E*)	24.39%
$\mathrm{Estimated}\;\mathrm{critical}\;\mathrm{age}\;(t_{ec}$) ^3^	5.05 a
The opening catch body length ^1^	130 mm
*E*_0.1_	0.35
*E*_50%_	0.28
*E* *_max_*	0.47
Burbot population	273,400
The biomass of burbot population ^4^	94.76 T

^1^ mm represents millimeters; ^2^ g represents gram; ^3^ a represents age; ^4^ T represents tons.

**Table 3:** Fishing pressure based on virtual population analysis of burbot in the study area.

Mid-Length	Catch n	Population n	Fishing Mortality F	Steady-State Biomass T
105	7.96	18,807.64	0.0031	0.22
115	15.92	18,000.71	0.0063	0.29
125	15.92	17,203.14	0.0065	0.37
135	87.58	16,423.09	0.0364	0.46
145	59.72	15,590.57	0.0255	0.56
155	67.68	14,806.14	0.0298	0.67
165	99.53	14,033.52	0.0451	0.79
175	99.53	13,249.85	0.0466	0.92
185	95.55	12,487.8	0.0462	1.06
195	155.26	11,751.27	0.0779	1.21
205	123.41	10,978.13	0.0644	1.36
215	75.64	10,260.67	0.041	1.52
225	59.72	9612.63	0.0335	1.69
235	91.56	9000.39	0.0533	1.86
245	99.53	8376.71	0.0604	2.04
255	151.28	7766.72	0.0963	2.2
265	179.15	7128.6	0.1206	2.35
275	95.55	6488.89	0.0679	2.5
285	135.36	5957.11	0.1015	2.65
295	95.55	5408.49	0.0759	2.79
305	91.56	4922.61	0.0769	2.92
315	71.66	4462.1	0.0637	3.06
325	79.62	4041.96	0.0752	3.18
335	55.74	3633.93	0.0559	3.29
345	59.72	3269.18	0.0637	3.39
355	43.79	2919.06	0.0498	3.47
365	51.75	2602.92	0.063	3.54
375	43.79	2296.6	0.0573	3.59
385	27.87	2016.03	0.0392	3.62
395	15.92	1767.92	0.024	3.66
405	7.96	1546.72	0.0129	3.69
415	7.96	1347.29	0.0139	3.71
425	23.89	1161.22	0.0453	3.67
435	35.83	973.84	0.0757	3.54
445	7.96	791.35	0.0188	3.4
455	27.87	652.09	0.0743	3.23
465	7.96	507.96	0.0243	3.02
475	19.91	398.56	0.0708	2.77
485	15.92	291.56	0.0687	2.44
495	19.91	203.82	0.1098	2.03
505	0	127.69	0	1.69
515	0	83.85	0	1.43
525	11.94	48.97	0.1	1.61

## Data Availability

Data are contained within the article.
